# Clinical features and outcome of eight patients with *Chlamydia psittaci* pneumonia diagnosed by targeted next generation sequencing

**DOI:** 10.1111/crj.13681

**Published:** 2023-08-08

**Authors:** Yanping Zhang, Xiangsen Jiang, Wei Ye, Jinlin Sun

**Affiliations:** ^1^ Department of Respiratory Medicine Shandong Provincial Third Hospital Jinan P.R. China; ^2^ Imaging Center Shandong Provincial Third Hospital Jinan P.R. China; ^3^ Department of Respiratory Medicine Hui Ya Hospital of The First Affiliated Hospital, Sun Yat‐Sen University Huizhou P.R. China

**Keywords:** *Chlamydia psittaci* pneumonia, community‐acquired pneumonia, imaging characteristics, laboratory examination, next‐generation sequencing

## Abstract

**Introduction:**

The clinical symptoms of *Chlamydia psittaci* pneumonia are still poorly understood. This study was designed to summarize the clinical features and outcome of eight *C. psittaci* pneumonia patients diagnosed by targeted next generation sequencing (tNGS).

**Methods:**

We included eight *C. psittaci* pneumonia patients admitted to our hospital from January 2021 to July 2022. The tNGS was performed to the samples collected from bronchoalveolar lavage fluid of each patient. Their clinical data were analysed, including baseline features, clinical symptoms, chest radiographic findings and laboratory examinations.

**Results:**

The tNGS sequence number for *C. psittaci* was in a range of 10 to 1722. The radiographic characteristics were mainly featured by patchy consolidation shadows, ground‐glass density shadows, air bronchogram and slight pleural effusion. Within 1–3 days after hospitalization, most patients showed increased neutrophil ratio, C‐reactive protein and erythrocyte sedimentation rate, and decreased lymphocyte count, total protein, albumin and prealbumin. Some patients showed increased glutamic‐pyruvic transaminase, glutamic‐oxaloacetic transaminase and lactate dehydrogenase levels. Three critically ill patients showed increased creatine kinase, creatine kinase isoenzyme and high‐sensitivity troponin T (hs‐TnT) levels.

**Conclusions:**

A poultry or bird contact history, typical flu‐like symptoms, patchy consolidation, ground‐glass density shadow and air bronchogram may contribute to the diagnosis and treatment of *C. psittaci* pneumonia. Increase in creatine kinase, creatine kinase isoenzyme and hs‐TnT may indicate a severe condition. Moxifloxacin and minocycline were effective in the management of *C. psittaci* pneumonia.

## Graphical abstract

The tNGS technique is of great value in the diagnosis and management of *C. psittaci* pneumonia. A poultry or bird contact history, typical flu‐like symptoms, radiographic characteristics such as patchy consolidation, ground‐glass density shadow and air bronchogram may contribute to the diagnosis of *C. psittaci* pneumonia. Moxifloxacin and minocycline are effective in the management of *C. psittaci* pneumonia.

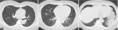

## 1 INTRODUCTION

*Chlamydia psittaci* pneumonia is a zoonosis caused by *C. psittaci* designated as a Gram‐negative and obligate intracellular parasites. Individuals are more likely to be infected upon direct contact with the infectious birds, poultry, mammals or their feces and respiratory secretions, as well as contaminated aerosols.^1^, ^2^, ^3^ *C. psittaci* pneumonia accounts for approximately 1.03% of community‐acquired pneumonia (CAP), with a large number of patients progressing into critically ill conditions.^4^, ^5^ To date, the differential diagnosis of *C. psittaci* pneumonia is in a dilemma due to its non‐specific clinical manifestations, yielding a high incidence of misdiagnosis and missed diagnosis. Therefore, it is crucial to understand the clinical phenotype of *C. psittaci* pneumonia for the early diagnosis of such disease.

Recent studies have reported the clinical features of *C. psittaci* pneumonia. In a multicentre observational study based on a cohort of 116 patients,^6^ Yang et al. summarized the laboratory indices, treatment options and risk factors of *C. psittaci* pneumonia. In addition, Wang et al. comprehensively discussed the clinical characteristics of *C. psittaci* pneumonia.^7^ Nevertheless, these studies have some limitations to some extent, such as not illustrating the chest computed tomography (CT) features or a small sample size.

Next generation sequencing (NGS) contributes to the rapid screening of pathogens, which facilitates the timely identification of pathogens and the treatment.^8^ Several studies have reported that the use of metagenomic NGS (mNGS) improved the diagnosis of *C. psittaci* pneumonia.^9^, ^10^ However, to the best of our knowledge, rare studies have focused on the diagnostic value of targeted NGS (tNGS) in *C. psittaci* pneumonia. Herein, we retrospectively analysed the clinical data of eight patients with *C. psittaci* pneumonia diagnosed by tNGS, including baseline features, physical signs, CT imaging, laboratory examination results and treatment outcomes.

## 2 MATERIALS AND METHODS

### 2.1 Patients

In this retrospective study, we included eight patients with *C. psittaci* pneumonia admitted to our hospital from January 2021 to July 2022. Inclusion criteria were *C. psittaci* pneumonia patients diagnosed by tNGS based on the samples collected from bronchoalveolar lavage fluid (BALF). All patients or their relatives signed informed consent forms. The study was approved by the ethical committee of our institution.

### 2.2 The tNGS procedures

The tNGS was performed to identify the pathogenic bacteria in patients with atypical pulmonary infection according to the operating procedures (KingMed Diagnostics, Hangzhou, China). Briefly, the clinical BALF samples were collected in line with standard aseptic processing procedures. Upon sample collection, the BALF samples were transferred by an uninterrupted cold‐chain transportation to a genetic testing company. The genetic testing was carried out according to the previous description,^8^, ^9^ with DNA library constructed by breaking the DNA fragment into 150–250 bp insert fragments, followed by terminal repair and adapter connection. Finally, generated data were classified through aligning to the Microbial Genome Databases, including viruses, fungi, bacteria and parasites.

### 2.3 Data collection

We collected the general information of each patient, including gender, age, underlying diseases, a poultry or bird contact history, disease onset time, occupation, smoking history and body mass index. Besides, the clinical manifestations, physical signs, CT imaging characteristics and laboratory examination results were also collected.

The CT imaging results included frequency of CT examination, lobular involvement, basic features of intrapulmonary lesions and extrapulmonary manifestations. All images were evaluated independently by two experienced imaging physicians. In cases of disputes between them, a detailed communication was held until consensus.

The laboratory data were collected on day 1 to 3 after hospitalization, day 4 to 7 after hospitalization and the day of discharge. The data were as follows: white blood cell (WBC) count, neutrophil count and ratio, lymphocyte count and ratio, C‐reactive protein (CRP), procalcitonin, alanine aminotransferase, aspartate aminotransferase, lactate dehydrogenase, erythrocyte sedimentation rate (ESR), total protein, albumin, prealbumin, glutamic‐pyruvic transaminase (GPT), glutamic‐oxaloacetic transaminase (GOT), lactate dehydrogenase, creatine kinase (CK), creatine kinase isoenzyme, creatinine and high‐sensitivity troponin T.

### 2.4 Treatment and follow‐up

After admission, Case 8 received piperacillin sodium‐tazobactam sodium, and the other seven patients were empirically given moxifloxacin injection for anti‐infection treatment. Some patients received piperacillin sodium‐tazobactam sodium or cefoperazone sodium‐sulbactam sodium in combination with moxifloxacin. After obtaining the pathogenic results, four cases received intravenous moxifloxacin combined with oral minocycline, two cases received intravenous azithromycin combined with oral minocycline, one case received levofloxacin sodium chloride injection combined with oral minocycline, and one case received oral minocycline alone due to persistent liver function damage. Patients were followed up within 1 month after treatment, and pulmonary CT results and laboratory indicators were obtained.

### 2.5 Statistical analysis

All data were analysed by SPSS software (23.0 version). Continuous variables normally distributed were presented as means ± standard deviations and were analysed using the Student's *t* test. Continuous variables that were not normally distributed were expressed as median and were analysed using *z* test. Categorical variables were expressed as number and percentage. The significance level was set at *P* < 0.05.

## 3 RESULTS

### 3.1 The tNGS findings

*C. psittaci* was detected in all the BALF samples. The tNGS sequence number for *C. psittaci* was in a range of 10 to 1722. In addition to *C. psittaci*, most of the other pathogens detected by the tNGS procedures were background bacteria or colonized bacteria showing no clinical significance (Table 1).

**TABLE 1:** Detailed information of tNGS results and reads.

Patient no.	Specimen	tNGS results and reads
1	BALF	*Chlamydia psittaci* (80)
2	BALF	*C. psittaci* (90); *Haemophilus haemolyticus* (165); *Streptococcus pneumoniae* (112); *Klebsiella pneumoniae* (22); *Candida albicans* (19).
3	BALF	*C. psittaci* (10); *K. pneumoniae* (19).
4	BALF	*C. psittaci* (65); *Human gammaherpesvirus 4* (478); *C. albicans* (19); *Enterobacter cloacae complex* (4); *Human alphaherpesvirus* 1 (6).
5	BALF	*C. psittaci* (351); *C. albicans* (678); *S. pneumoniae* (113); *Staphylococcus aureus* (69); *Haemophilus influenzae* (52); *Human betaherpesvirus* 7 (3).
6	BALF	*C. psittaci* (278); *C. albicans* (958); *Human alphaherpesvirus 1* (108); *Moraxella catarrhalis* (11); *Human betaherpesvirus 7* (7).
7	BALF	*C. psittaci* (1722); *Aspergillus fumigatus* (9536); *C. albicans* (21537); *Neisseria meningitidis* (1091); *H. influenzae* (31994); *Acinetobacter baumannii* (72).
8	BALF	*C. psittaci* (24); *Veillonella parvula* (10724); *C. albicans* (1239); *Streptococcus mitis* (199); *S. pneumoniae* (153); *Campylobacter concisus* (31); *Mycoplasma salivarium* (334).

Abbreviation: BALF, bronchoalveolar lavage fluid.

### 3.2 Baseline features and clinical manifestations

Among 8 patients, there were 4 males and 4 females with an age range of 31–67 years. The onset time of disease was distributed in January (Case 6), April (Case 7), May (Cases 2 and 5), June (Cases 3, 4 and 8) and July (Case 1). Seven patients (87.5%) had a poultry or bird contact history, and the rest one patient (12.5%) had underlying chronic diseases. All the patients showed no digestive tract symptoms except Case 1 with dull pain in the lower abdomen (Table 2). All patients showed fever, with the peak value median of 39.55°C (range 38.7–40.5°C). Six (75%) patients showed intolerance of cold, seven (87.5%) cases showed cough, and five (62.5%) cases showed muscle soreness. Four (50%) patients showed expectoration and fatigue, respectively. Sore throat occurred in two cases (25.0%). Most (87.5%) cases showed general or poor appetite (Table 3).

**TABLE 2:** Baseline characteristics and clinical manifestations of eight patients with *C. psittaci* pneumonia.

Case	Gender	Age (years)	Disease onset time (month)	Poultry or bird contact history	Previous medical history	Occupation	Smoking history	BMI	Clinical manifestations
1	Female	33	7	Raising pigeons	No	Teacher	No	25.21	Fever for 2 days with maximum (max) temperature of 40.5°C, intolerance of cold, chills, cough, chest tightness, dull pain in the lower abdomen
2	Male	59	5	Raising chickens, and the chickens died of unknown reasons recently	Coronary heart disease and hypertension	Farmer	No	31.88	Fever for 7 days with max temperature of 39.6°C, intolerance of cold, chills, muscle soreness, poor appetite
3	Male	61	6	Raising parrots	No	Unoccupied	Smoking for 20 years, 40 cigarettes/day; quit smoking for 20 yrs	20.57	Fever for 30 days with max temperature of 38.7°C, cough, expectoration, sore throat, general appetite
4	Female	61	6	Raising parrots	No	Unoccupied	No	22.31	Fever for 10 days with max temperature of 39.7°C, intolerance of cold, cough (mostly dry cough), expectoration, chest tightness, fatigue, headache, chest pain, muscle soreness, general appetite
5	Female	31	5	No	No	Corporate employee	No	21.48	Fever for 3 days with max temperature of 39.5°C, intolerance of cold, cough, chest tightness, fatigue, headache, sore throat, muscle soreness, general appetite
6	Male	56	1	Raising ducks	No	Worker at a duck slaughtering plant	Smoking for 10 years, 15 cigarettes/day	23.88	Fever for 8 days with max temperature of 40.0°C, intolerance of cold, chills, cough, chest tightness, fatigue, muscle soreness, general appetite
7	Female	59	4	Raising parrots	No	Retiree	No	24.65	Fever for 3 days with max temperature of 39.5°C, intolerance of cold, chills, cough, expectoration, fatigue, headache, muscle soreness, general appetite
8	Male	67	6	Raising pigeons	No	Unoccupied	No	23.22	Fever for 1 day with max temperature of 38.7°C, cough, less sputum (not easy to cough up), general appetite

Abbreviation: BMI, body mass index.

**TABLE 3:** Number and percentage of patients with main clinical symptoms.

Fever	Intolerance of cold	Cough	Muscle soreness	Expectoration	Fatigue	Sore throat	General or poor appetite
8 (100%)	6 (75.0%)	7 (87.5%)	5 (62.5%)	4 (50.0%)	4 (50.0%)	2 (25.0%)	7 (87.5%)

### 3.3 CT imaging characteristics at diagnosis

**FIGURE 1:**
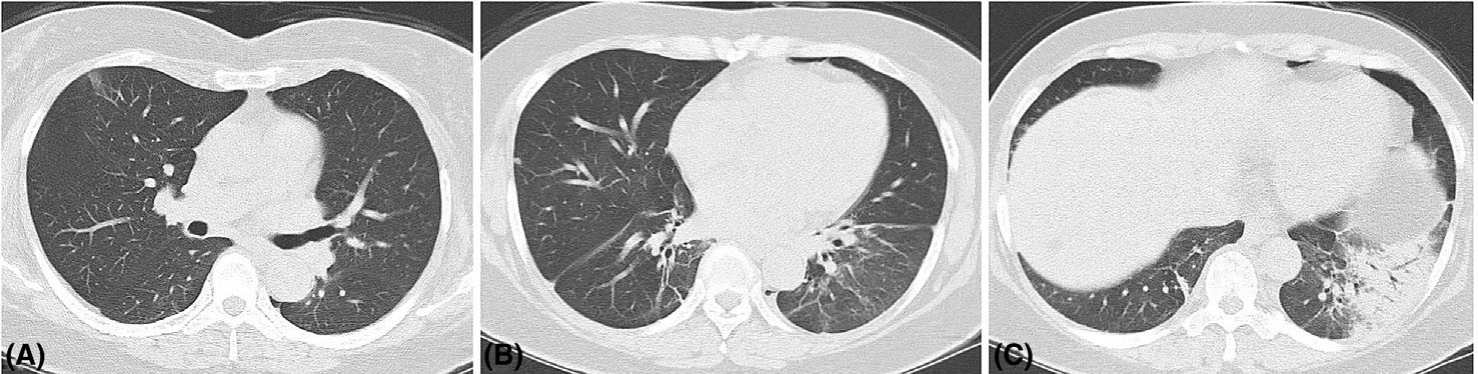
CT findings of a typical case (Case 7, female, 59 years old). (A) Small patchy ground‐glass density shadows in the subpleural right lung upper lobe. (B) Small patchy consolidation shadows in the subpleural right lung middle lobe. (C) Consolidations, ground‐glass density shadows, and air bronchogram in the left lung inferior lobe, and consolidation shadows in the right lung inferior lobe.

Early‐stage intrapulmonary lesions mainly involved a single lobe of the lung. Lesions were small in scope and common in the subpleura. Some lesions were fan‐shaped or wedge‐shaped outward from the hilum of the lung, presenting small patchy consolidation shadows or ground‐glass density shadows. Several consolidations were accompanied by ground‐glass density shadows. Air bronchogram appeared in some CT images. The inferior lobe of the lung was the most involved, especially the left inferior lobe. Most patients showed unilateral pleural effusion (Table 4). One patient (Case 1) showed ameliorative lesions in the left lung inferior lobe in the second chest CT scan. In contrast, the other seven patients (Cases 2–8) showed progression in intrapulmonary lesions, increased lesions with extended range, involvement of multiple pulmonary lobes, and expanded consolidations. In addition, most lesions were accompanied by air bronchogram (Figure 1), which showed increased pleural effusion or bilateral pleural effusion. Eight patients received 22 CT examinations in total, in which 16 CT (72.7%) displayed the presence of consolidation shadows, 22 (100%) displayed ground‐glass density shadows, and 16 (72.7%) showed air bronchogram. Seven patients (87.5%) showed a small amount of pleural effusion at single side. Cases 3 and 5 showed a small amount of bilateral pleural effusion in the second examination. Hilar and mediastinal lymphadenectasis was observed in Cases 7 and 8, respectively. No patients showed splenomegaly.

**TABLE 4:** Chest CT imaging characteristics of eight patients with *C. psittaci* pneumonia.

Case	CT examination	Involved lung lobe	Consolidation	Ground‐glass density shadow	Air bronchogram	Pleural effusion	Lymphadenectasis	Splenomegaly
1	1st	I (L)	+	+	+	Small amount (L)	−	−
	2nd	I (L)	−	+	−	−	−	−
2	1st	U and M (R) U and I (L)	+	+	+	−	−	−
	2nd	U (R) I (L)	−	+	−	−	−	−
3	1st	U, M and I (R) I (L)	+	+	+	Small amount (R)	−	−
	2nd	U, M and I (R) I (L)	+	+	+	Small amount (L and R)	−	−
4	1st	M and I (R)	+	+	+	Small amount (R)	−	−
	2nd	M and I (R)	−	+	−	−	−	−
5	1st	U (R)	+	+	+	−	−	−
	2nd	U, M and I (R) U and I (L)	+	+	+	Small amount (L and R)	−	−
	3rd	U, M and I (R) U and I (L)	+	+	+	Small amount (L and R)	−	−
	4th	U and I (R) U and I (L)	−	+	−	−	−	−
6	1st	I (L)	+	+	+	Small amount (L)	−	−
	2nd	U, M and I (R) U and I (L)	+	+	+	Small amount (L)	−	−
	3rd	U, M and I (R) U and I (L)	+	+	+	Small amount (L)	−	−
	4th	U, M and I (R) U and I (L)	+	+	+	−	−	−
			−	+	−	−	−	−
7	1st	I (L)	+	+	+	−	+	−
	2nd	U and I (R) I (L)	+	+	+	Small amount (L)	+	−
	3rd	I (R) I (L)	−	+	−	−	+	−
8	1st	I (L)	+	+	+	−	+	−
	2nd	U and I (L)	+	+	+	Small amount (L)	+	−
	3rd	U and I (L)	+	+	+	−	+	−

U, M and I represent the upper, middle and inferior lung lobes, respectively. L and R represent the left and right lungs, respectively.

### 3.4 Laboratory indicators at diagnosis

Most patients showed normal counts of WBC and neutrophil within 1 to 3 days after hospitalization, except two cases with increased WBC count (Case 4, 11.7 × 10^9^/L; Case 6, 12.4 × 10^9^/L) and three cases with increased neutrophil count (Case 4, 10.03 × 10^9^/L; Case 6, 11.61 × 10^9^/L; Case 7, 6.97 × 10^9^/L). Six patients showed increased neutrophil ratio. All patients presented increased CRP and ESR, and three cases presented increase in procalcitonin (Case 2, 2.51 ng/mL; Case 5, 2.28 ng/mL; Case 6, 4.15 ng/mL). Six cases showed decreased lymphocyte count and 7 cases showed decreased lymphocyte ratio. Biochemical tests showed increased levels of GPT, GOT, lactate dehydrogenase, CK and creatine kinase isoenzyme in some patients (4, 4, 6, 3 and 3 cases, respectively). Most patients showed hypoalbuminemia with decreased levels of total protein, albumin and prealbumin (7, 7 and 8 cases, respectively). All indices tended to be normal within 4 to 7 days after hospitalization and before discharge. However, the CRP level in five patients was still higher than the normal value. All patients showed no improvement in ESR. Total protein, albumin and prealbumin levels remained low in almost all patients within 7 days after hospitalization, and prealbumin level showed increase before discharge. GPT, GOT and lactate dehydrogenase were always at a high level from hospitalization to discharge (Tables 5, 6, 7).

**TABLE 5:** Changes in each laboratory indicator from hospitalization to discharge among eight patients.

Case	Indicator													
	WBC count (×10^9^/L)	Neutrophil count (×10^9^/L)	Lymphocyte count (×10^9^/L)	Lymphocyte ratio (%)	CRP (mg/L)	PCT (ng/mL)	Albumin (g/L)	Prealbumin (mg/L)	GOT (U/L)	GPT (U/L)	Lactate dehydrogenase (U/L)	CK (U/L)	Creatine kinase isoenzyme (ng/mL)	Creatinine (μmol/L)
1–3 days after hospitalization														
1	4.85	3.07	1.26	25.91	76.9	0.24	36.3	147	54.6	69.7	297	181	3.69	181
2	6.8	5.5	0.96	14.12	153.8	2.51	32.2	116	54	76	247	253	4.47	253
3	4.99	4.01	0.74	14.83	87.8	0.09	40.1	107	20.6	21.4	269	143	1.68	143
4	11.7	10.03	0.95	8.1	142.6	0.38	29.1	164	13.8	67.2	201	22	2.2	22
5	5.8	4.67	0.93	16.03	164.2	2.28	31.2	102	199.2	186.1	921	1985.2	46.92	1985.2
6	12.4	11.61	0.33	2.66	181.1	4.15	37.4	21	127.2	73.2	532	3802.5	12.12	3802.5
7	8.42	6.97	0.74	8.8	123.3	0.19	35	154	49.3	31.3	240	276	4.51	276
8	6.35	4.23	0.99	15.59	72.3	0.12	35.9	87	26.1	15.3	230	77.14	0.18	77.14
4–7 days after hospitalization														
1	5.82	3.26	1.82	31.25	12.7	0.11	34.1	223	81.9	131.6	282	72	4.25	72
2	4.31	2.83	1	23.2	115	0.4	35.6	242	34.1	48.3	258	106	4.08	106
3	3.69	2.21	1.12	30.33	54.5	0.31	36.4	156	44	34	330	205	0.67	205
4	7.51	5.67	0.94	12.52	146.2	0.26	30.2	115	18.3	35.6	193	24	1.44	24
5	5.1	4.2	0.66	12.94	114.9	0.47	29.6	106	168.6	217	778	256.7	4.58	256.7
6	8.9	7.99	0.47	5.28	168.7	1.71	39.8	69	489.4	336.5	783	2364	1.99	2364
7	7.85	5.6	1.58	20.12	27.6	0.04	34.1	169	46.2	36.4	214	86	2.15	86
8	3.76	2.5	1.04	13.25	116.9	0.02	34.4	151	20.4	20.7	221	73	0.88	73
Before discharge														
1	5.86	4.73	2.39	40.75	3.1	0.04	40.2	226	22.3	85.2	164	52	1.96	52
2	8.8	5.47	2.1	24.3	3.4	0.02	38.6	269	18	24.2	217	55	1.79	55
3	4.56	2.25	1.11	24.34	11.1	0.01	38.2	228	20.3	22.6	255	62	1.82	62
4	5.2	3.08	1.27	24.34	7.7	0.04	32.3	193	20.7	27.6	175	35	2.2	35
5	6.22	2.56	2.72	43.73	5.2	0.07	36.6	228	47	72.3	404	60	2.91	60
6	6.39	3.82	1.12	17.52	29	0.05	40.5	127	36.6	57.2	226	95	1.67	95
7	7.95	5.8	1.68	21.13	27.8	0.02	40.1	234	40.2	36.8	213	102	3.31	102
8	5.68	4.16	1.27	14.69	36.8	0.02	40.2	‐	‐	15.2	216	52	0.97	52

Abbreviations: CK, creatine kinase; CRP, c‐reactive protein; GOT, glutamic‐oxaloacetic transaminase; GPT, glutamic‐pyruvic transaminase; PCT, procalcitonin; WBC, white blood cell.

**TABLE 6:** The number of patient(s) with changes in each laboratory indicator from hospitalization to discharge.

Change in examination index	Number of patient(s) within 1–3 days after hospitalization	Number of patient(s) within 4–7 days after hospitalization	Number of patient(s) before discharge
WBC count ↑	2	0	1
Neutrophil count ↑	3	2	1
Neutrophil ratio ↑	6	3	2
Lymphocyte count ↓	6	6	2
Lymphocyte ratio ↓	7	5	3
CRP ↑	8	8	5
PCT ↑	3	1	0
ESR ↑	8	8	8
Total protein ↓	7	7	5
Albumin ↓	7	8	7
Prealbumin ↓	8	8	2
GPT ↑	4	4	4
GOT ↑	4	4	2
Lactate dehydrogenase ↑	6	6	4
CK ↑	3	4	1
Creatine kinase isoenzyme ↑	3	0	0
Creatinine ↑	1	0	0
hs‐TnT ↑	3	3	3

*Note*: ↑ represents increase, and ↓ represents decrease.

Abbreviations: CK, creatine kinase; CRP, c‐reactive protein; ESR, erythrocyte sedimentation rate; GOT, glutamic‐oxaloacetic transaminase; GPT, glutamic‐pyruvic transaminase; hs‐TnT, high‐sensitivity troponin T; PCT, procalcitonin; WBC, white blood cell.

**TABLE 7:** Values [median (minimum to maximum)] of each laboratory examination index of patients from hospitalization to discharge.

Examination index	Values within 1–3 days after hospitalization	Values within 4–7 days after hospitalization	Values before discharge
WBC count (×10^9^/L)	6.80 (4.99–12.40)	5.46 (3.69–9.17)	5.79 (3.75–9.95)
Neutrophil count (×10^9^/L)	5.60 (3.58–11.61)	3.73 (2.21–7.99)	3.76 (2.02–8.16)
Neutrophil ratio (%)	82.3 (70.44–93.6)	71.15 (56.09–89.8)	66.75 (44.2–81.89)
Lymphocyte count (×10^9^/L)	0.69 (0.33–1.27)	0.88 (0.47–1.82)	1.20 (1.04–2.39)
Lymphocyte ratio (%)	8.9 (2.7–24.95)	15.89 (5.3–31.25)	24.12 (10.48–48.9)
CRP (mg/L)	146.83 (72.3–181.1)	130.9 (12.7–168.7)	9.4 (3.1–36.4)
PCT (ng/mL)	0.42 (0.09–4.15)	0.21 (0.02–1.71)	0.035 (0.01–0.08)
ESR (mm/h)	59 (40–104)	42.5 (22–105)	28.5 (21–61)
Total protein (g/L)	59.85 (51.7–68.3)	56.9 (49.0–66.9)	64.55 (56.4–73.1)
Albumin (g/L)	34.05 (28.6–40.1)	30.8 (24.7–37.5)	37.35 (29.2–42.3)
Prealbumin (mg/L)	90.5 (17.3–164.0)	103.0 (21–223)	241.5 (127–284)
GPT (U/L)	47.1 (15.3–186.1)	41.95 (20.7–336.5)	34.9 (13.6–85.2)
GOT (U/L)	35.80 (13.80–127.2)	39.55 (18.8–189.4)	21.5 (13.2–55.7)
Lactate dehydrogenase (U/L)	274 (186–921)	306 (193–783)	223.5 (164–447)
CK (U/L)	170 (22–3802.5)	155.5 (20–2364)	53.5 (35–888)
Creatine kinase isoenzyme (ng/mL)	3.76 (1.34–46.92)	2.57 (0.88–4.58)	1.82 (0.98–2.29)
Creatinine (μmol/L)	62.25 (46.2–85.5)	55.5 (45–75)	53 (44–80)
hs‐TnT (ng/mL)	17.13 (10.87–246.1)	16.64 (11.57–290.1)	15.57(13.56–248.2)

*Note*: The normal ranges of each index are as follows: WBC (×10^9^/L), 3.5–9.5; neutrophil count (×10^9^/L), 1.8–6.3; neutrophil ratio (%), 40–75; lymphocyte count (×10^9^/L), 1.1–3.2; lymphocyte ratio (%), 20–50; CRP (mg/L), 0–6; PCT (ng/mL), 0–0.5; ESR (mm/h), 0–20; total protein (g/L), 65–85; albumin (g/L), 40–55; prealbumin (mg/L), 180–350; GPT (U/L), 7–40; GOT (U/L), 15–35; lactate dehydrogenase (U/L), 120–250; CK (U/L), 40–200; creatine kinase isoenzyme (ng/mL), 0–5; creatinine (μmol/L), 45–84; and hs‐TnT (ng/mL), 0–14.

Abbreviations: CK, creatine kinase; CRP, c‐reactive protein; ESR, erythrocyte sedimentation rate; GOT, glutamic‐oxaloacetic transaminase; GPT, glutamic‐pyruvic transaminase; hs‐TnT, high‐sensitivity troponin T; PCT, procalcitonin; WBC, white blood cell.

### 3.5 Treatment outcome

**FIGURE 2:**
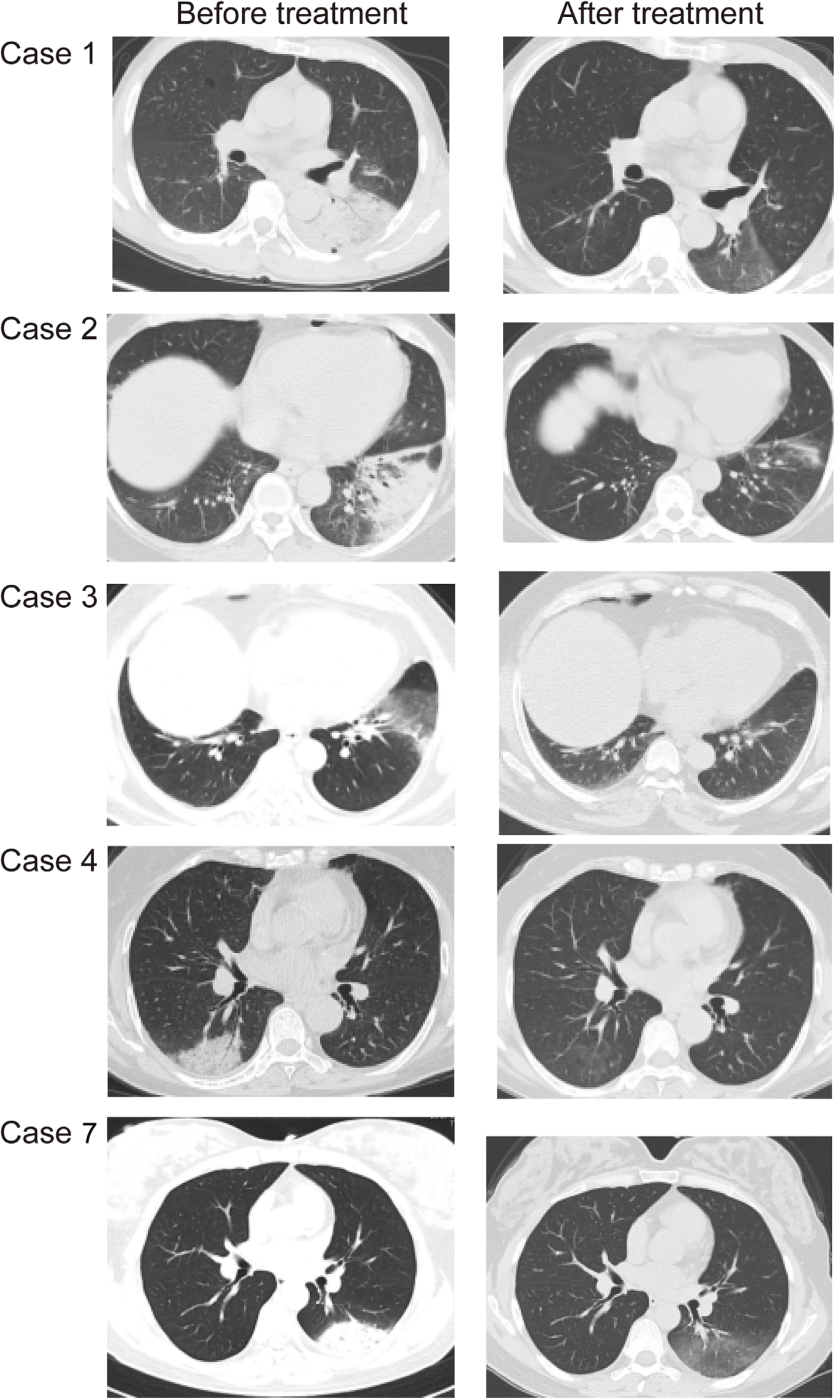
Pulmonary CT findings among the five patients before and after treatment.

Two cases (Cases 5 and 6) received only telephone follow‐up. Case 5 claimed to have relieved respiratory symptoms without recurrence. Case 6 had no fever, alleviated cough and sputum and recovered muscle strength (Table 8). Five cases (Cases 1, 2, 3, 4 and 7) underwent pulmonary CT re‐examination (Figure 2). Cases 1 and 2 showed consolidation shadows and air bronchogram in the left lung inferior lobe before treatment. After treatment, flocculent cord‐like high‐density shadows were found in the left lung, suggesting significant relief in the pneumonia. Compared with flaky‐ and club‐shaped high‐density shadows before treatment, Case 3 showed almost completely absorbed lesions after treatment. Case 4 showed patchy ground‐glass high‐density shadows and integrant consolidation shadows in the right lung middle and inferior lobes, accompanied by slight left pleural effusion before treatment. After treatment, only scattered ground‐glass shadows were found in right lung without consolidation shadows, and the pleural effusion in the left lung was completely absorbed. Case 7 presented patchy high‐density shadows and some consolidation shadows in the left lung inferior lobe, as well as a small amount of left pleural effusion before treatment. Notably absorbed lesions and completely absorbed pleural effusion were found after treatment.

**TABLE 8:** Therapeutic schedules and outcomes of eight patients.

Case	Time interval from admission to diagnosis (days)	Hospital stay (days)	Initial therapeutic schedule	Outcome after initial treatment	Post‐diagnosis schedule	Post‐discharge medication	Time interval from diagnosis to antibiotics discontinuation (days)	Outcome
1	7	15	Moxifloxacin + piperacillin sodium tazobactam sodium	Poor outcome (fever after 72 h, PCT > 2 ng/mL, CRP > 100 mg/mL). The initial schedule was discontinued and replaced with meropenem.	Withhold meropenem; Intravenous azithromycin plus oral minocycline	Oral azithromycin and minocycline	22	Lung CT showed obvious pneumonia absorption after 28 days
2	3	14	Moxifloxacin + cefoperazone sodium sulbactam sodium	No fever; CRP was decreased by 25%, still >100 mg/mL	Intravenous azithromycin plus oral minocycline	Oral azithromycin and minocycline	25	Lung CT showed obvious pneumonia absorption after 20 days
3	4	13	Moxifloxacin	No fever; CRP and PCT were decreased; ALT and AST were increased	Oral minocycline; Moxifloxacin was discontinued due to persistent abnormal liver function	Oral minocycline	23	Lung CT showed complete pneumonia absorption after 34 days
4	3	8	Moxifloxacin + piperacillin sodium tazobactam sodium	No fever; CRP was decreased; PCT was increased	Moxifloxacin plus oral minocycline	Oral moxifloxacin and minocycline	19	Lung CT showed obvious pneumonia absorption after 15 days
5	3	10	Moxifloxacin + piperacillin sodium tazobactam sodium	No fever; cough and sputum were relieved; CRP was increased; PCT was decreased	Moxifloxacin plus oral minocycline	Oral moxifloxacin and minocycline	21	Telephone follow‐up, the respiratory symptoms were relieved with no recurrence after 20 days
6	3	10	Moxifloxacin + piperacillin sodium tazobactam sodium	Poor outcome (Fever, CRP > 100 mg/mL). The initial schedule was discontinued and replaced with meropenem.	Moxifloxacin plus oral minocycline	Oral moxifloxacin and minocycline	21	Telephone follow‐up, cough and sputum were alleviated, and muscle strength was recovered with no fever after 20 days
7	3	8	Moxifloxacin	Poor outcome (Fever after 72 h, CRP > 100 mg/mL)	Moxifloxacin plus oral minocycline	Oral moxifloxacin and minocycline	18	Lung CT showed obvious pneumonia absorption after 18 days
8	4	8	Piperacillin sodium tazobactam sodium 4.5 gq8h	Recurrent fever, CRP was slightly decreased	Levofloxacin sodium chloride injection 0.5 gqd plus oral minocycline	Oral minocycline	21	Almost complete pneumonia absorption

Besides, a total of six cases underwent laboratory examination after treatment. Significant increase was seen in the lymphocyte count (0.81 to 1.69). In addition, significant decrease was observed in the levels of CRP (148.2 to 6.45), CK (217 to 7.5) and lactate dehydrogenase (283 to 221.5) (*P* < 0.05, Table 9).

**TABLE 9:** Changes of laboratory indicators before and after treatment in six patients followed up.

Indicators	Before treatment (*n* = 6)	After treatment (*n* = 6)	*t/z* test	*P* value
Lymphocyte count (×10^9^/L)	0.81 ± 0.32	1.69 ± 0.69	−3.99	0.01
CRP (mg/L)	148.2 (85.08, 168.43)	6.45 (3.33, 15.58)	−2.2	0.03
PCT (ng/mL)	1.6083 ± 1.64	0.04 ± 0.02	2.36	0.07
ALT (U/L)	71.45 (55.75, 103.53)	43.35 (26.75, 75.53)	−1.57	0.12
AST (U/L)	78.23 ± 71.62	27.42 ± 11.7	2.06	0.1
CK (U/L)	217 (112.75, 2439.53)	7.5 (47.75, 70.25)	−1.99	0.04
LDH (U/L)	283 (235.5, 629.25)	221.5 (172.25, 292.25)	−2.2	0.03

ALT, alanine aminotransferase; AST, aspartate aminotransferase; CRP, C‐reactive protein; CK, creatine kinase; LDH, lactate dehydrogenase; PCT, procalcitonin.

## 4 DISCUSSION

The diagnosis of *C. psittaci* pneumonia is still in a dilemma due to vague symptoms. Nowadays, its pathogenesis is still not well defined. In recent years, NGS contributes to the diagnosis of *C. psittaci* pneumonia, yielding improvement in the diagnosis and outcome of such disease. The mNGS is the major method for the detection of *C. psittaci* recently. In contrast, few studies have reported the application of tNGS in the detection of *C. psittaci*. The tNGS technique screens clinical isolates through a set of known pathogen sequences, which can detect more pathogenic microorganisms including bacteria, fungi and viruses quickly and objectively. It can also target pathogens known to be associated with specific diseases such as gastrointestinal or respiratory diseases.^11^, ^12^, ^13^ Besides, tNGS technology combines the advantages of PCR and NGS. It positively enriches the target pathogen through super multiplex PCR, which can eliminate host nucleic acid interference and improve detection sensitivity. Especially, compared with mNGS, tNGS has the advantages of rapidity, high sensitivity and high specificity, and its cost is only a quarter of that of mNGS. In this study, *C. psittaci* pneumonia was diagnosed by tNGS technique, which may provide insights into the application of tNGS in the diagnosis of *C. psittaci* pneumonia.

The clinical manifestations of *C. psittaci* pneumonia are non‐specific. Previous study had shown that people infected with *C. psittaci* often presented with influenza‐like atypical symptoms, such as fever, chills, headache, myalgia and fatigue, with or without respiratory symptoms.^14^ The disease could progress to severe pneumonia, and most cases were curable with few dead cases.^15^, ^16^, ^17^, ^18^ Analogously, all patients in our study showed fever, with the peak value median of 39.55°C (range 38.7–40.5°C). Seven (87.5%) cases showed cough, and five (62.5%) cases showed muscle soreness. Four (50%) cases showed expectoration and fatigue, respectively. Additionally, although three patients in this study developed severe pneumonia, they were cured with no death events.

Studies have shown that the radiographic features of *C. psittaci* pneumonia were mainly patchy consolidation shadows or ground‐glass density shadows, bronchiectasis, involvement in one or both lungs, inflammatory effusion of one or more lobes and partial pleural effusion. Besides, lesions could occur in any lobe, mainly in the inferior lobe and unilateral lung, particularly the right lung, and hilar lymphadenectasis was uncommon.^19^, ^20^, ^21^, ^22^ Broadly consistent but slightly different, lesions tended to occur in the left lung inferior lobe in our study, which may be related to small sample size. In addition, we also found that the early‐stage lesions were small in scope and more common in the subpleura. With the progression of lesions, multiple lobes were involved and increased lesions with extended range were observed. Consolidations also showed increased ranges with some fan‐shaped or wedge‐shaped outward from the hilum of the lung. Most lesions were accompanied by air bronchogram, which was consistent with previous finding that the presentation of the lesion was closely related to the severity of the disease.^23^

It has been reported that *C. psittaci* could proliferate rapidly after colonizing the respiratory tract, resulting in insignificant increase in leukocytes, CRP and ESR in the early stages. Subsequently, *C. psittaci* invaded the reticuloendothelial cells in the liver and spleen, resulting in changes in liver enzymes in some patients.^24^ Consistently, most patients in this study showed normal WBC and neutrophil count within 1 to 3 days after hospitalization. All patients showed increased neutrophil ratio, CRP and ESR. Besides, most patients showed increased levels of GPT, GOT, lactate dehydrogenase, CK and CK isoenzyme. In addition, most patients showed significantly reduce in lymphocyte count and lymphocyte ratio, together with varying degrees of reductions in total protein, albumin and prealbumin. Yang et al. found that lymphocytopaenia occurred in 93% of patients in critical condition and proposed that lymphocytopaenia was an important manifestation of patients with severe community‐acquired infections.^24^ This may be related to the fact that some atypical types of pneumonia destroy the cytoplasmic components of lymphocytes, leading to lymphocyte apoptosis. However, hypoalbuminemia presented in most patients in this study, which has been reported in a few studies.^20^ It may be associated with decreased protein intake, decreased albumin production induced by liver injury and increased albumin consumption. Furthermore, studies have shown that rhabdomyolysis and cardiac and renal impairment may occur in critically ill patients. Similarly, three critically ill patients in this study showed various increase in CK, CK isoenzyme and high‐sensitivity troponin T. In some cases, the recovery in laboratory examination indices before discharge were delayed than that in clinical manifestations, while some patients' condition showed improvement before discharge compared with the time of admission, with laboratory examination indices tended to normal values.

Tetracyclines, macrolides and quinolones have been shown to be effective in the treatment of *C. psittaci* pneumonia. Among them, tetracyclines are first‐line drugs against *C. psittaci* infection in human beings.^25^ Consistently, all patients in our study were sensitive to oral minocycline. Studies have shown that quinolones were less effective than tetracyclines and macrolides.^8^ Congruously, empirical administration of a quinolone (moxifloxacin) prior to diagnosis did not result in significant disease improvement in all patients in our study. After clarifying the aetiology by tNGS, we adjusted the treatment schedule. The clinical symptoms and laboratory indicators were significantly improved after the addition of tetracyclines. This confirmed the great value of tNGS in *C. psittaci* management. In contrast, study has reported that patients with *C. psittaci* pneumonia showed a good prognosis after taking quinolones alone.^26^ In the future, more clinical studies are needed to verify the therapeutic effect of these drugs on *C. psittaci* pneumonia.

There were some limitations in this study. First, the sample size was too small. Second, critically ill patients were not subjected to independent observation and analysis. In addition, imaging manifestations and laboratory index changes of advanced disease were uncertain due to the short‐term follow‐up. Studies base on a sufficiently large sample size and a long‐term follow‐up are necessary in the future.

## 5 CONCLUSION

In conclusion, tNGS was of great value in the diagnosis and management of *C. psittaci* pneumonia. Attention should be paid to the possibility of early‐stage *C. psittaci* pneumonia in cases of influenza‐like symptoms, a poultry or bird contact history and CT imaging characteristics such as patchy consolidation, ground‐glass density shadow and air bronchogram, together with various abnormal laboratory examination indices. Moxifloxacin and minocycline were effective in the management of *C. psittaci* pneumonia.

## AUTHOR CONTRIBUTIONS

Yanping Zhang analysed the data and drafted the manuscript. Xiangsen Jiang analysed the data and drafted the manuscript. Wei Ye collected the data. Jinlin Sun conceived the study design and revised the manuscript. All authors read and approved the final submission. Yanping Zhang and Xiangsen Jiang contributed equally to this work and shared the first authorship.

## CONFLICT OF INTEREST STATEMENT

The authors have no conflicts of interest to declare.

## ETHICS STATEMENT

The study was conducted in accordance with the Declaration of Helsinki (as revised in 2013). The study was approved by the Ethical Committee of Shandong Provincial Third Hospital. All patients or their relatives signed informed consent forms.

## Data Availability

The data used to support the findings of this study are available from the corresponding author upon request.
